# Editorial: Fluoride across the world: innovations, access, outcomes and controversies

**DOI:** 10.3389/froh.2026.2005477

**Published:** 2026-10-26

**Authors:** Marco Dettori, Peter Lingström, Guglielmo Campus

**Affiliations:** 1 Department of Medicine, Surgery and Pharmacy, University of Sassari, Sassari, Italy; 2 Department of Cariology, Sahlgrenska Academy, Institute of Odontology, University of Gothenburg, Gothenburg, Sweden; 3 Department of Oral and Maxillofacial Sciences, Sapienza University of Rome, Roma, Italy

**Keywords:** confidence, fluoride, hesitancy, public health, risk communication, social listening

## 1 Introduction: from evidence to confidence

Fluoride hesitancy, particularly regarding topical fluoride, has emerged as a practical communication challenge for oral-health professionals. Survey data have shown that dentists perceive caregiver fluoride refusal as a relevant and growing challenge, with a substantial proportion reporting discomfort when discussing fluoride with caregivers who refuse it (1). Qualitative research has further shown that oral health professionals use heterogeneous approaches when managing fluoride-hesitant caregivers, including education, insistence, disengagement and shared engagement, and that these approaches are not consistently grounded in evidence-based communication principles (2). These observations point to a problem that cannot be reduced to the availability of scientific information. Although much of the current empirical evidence concerns professionally applied topical fluoride, the proposed framework is intended to be applicable across fluoride-delivery contexts, with context-specific adaptation.

Fluoride therefore provides a useful case study in a broader contemporary public-health problem: scientific maturity does not automatically produce stable public confidence. Evidence about efficacy, exposure and safety remains essential, but decisions are also shaped by perceived control, trust in professionals and institutions, prior experiences, values, social narratives and the information environment. The practical question is no longer only what the evidence says. It is also how that evidence is interpreted, by whom, in which context and with what consequences for behaviour.

This Perspective paper argues that fluoride communication should be approached as an intervention requiring diagnosis, targeting and evaluation rather than as the simple transmission of facts. The authors argue that the next phase of fluoride research should move beyond documenting hesitancy towards identifying its determinants and prospectively testing responses. The Authors propose a diagnosis-driven framework designed for researchers, oral-health professionals, public-health authorities, professional organisations and communication teams. Its purpose is not to prescribe a single message or to classify people as being “for” or “against” fluoride. Its purpose is to define the problem before choosing the response, and to make the response empirically testable. The Authors use fluoride confidence to refer broadly to the conditions that enable individuals and populations to make informed decisions about fluoride based on credible evidence, an appropriate understanding of benefits, risks and uncertainty, and trust in the sources and processes through which that evidence is communicated. Confidence should therefore not be understood simply as the inverse of hesitancy, nor as an expectation that all individuals will reach the same decision. In this framework, hesitancy denotes uncertainty or ambivalence, whereas opposition refers to an explicitly negative stance toward fluoride use. These attitudinal constructs should also be distinguished from behavioural outcomes such as acceptance, delay or refusal, and from trust, which may influence confidence without being synonymous with it.

## 2 Why generic communication is insufficient

### 2.1 Fluoride hesitancy is heterogeneous

The statement “I do not want fluoride” may conceal very different underlying concerns. A caregiver may believe fluoride is unnecessary, may wish to avoid substances perceived as “chemicals”, may fear harm, may be uncertain about the quality or completeness of the evidence, or may distrust the professional relationship or feel pressured. These positions are not minor variants of the same attitude. They represent different mechanisms and therefore imply different communication tasks. Additional fluoride-specific determinants that warrant empirical investigation include perceptions of natural versus artificial interventions, commercial distrust related to dental products, previous dental experiences, differing understandings of caries as a disease process, and exposure to social-media narratives promoting non-fluoride alternatives.

A knowledge-deficit response assumes that reluctance is primarily caused by insufficient factual knowledge and that additional information should therefore reduce reluctance. This may be appropriate when the barrier is a correctable misunderstanding about benefit, dose or exposure. It is less plausible when the dominant barrier is distrust, perceived coercion, identity, loss of control or an unresolved uncertainty that the person regards as legitimate. In those circumstances, supplying more facts without addressing the actual mechanism may be ineffective and, in some circumstances, counterproductive. The overall diagnosis-driven framework and action cycle are summarized in Figure 1.

**Figure 1:**
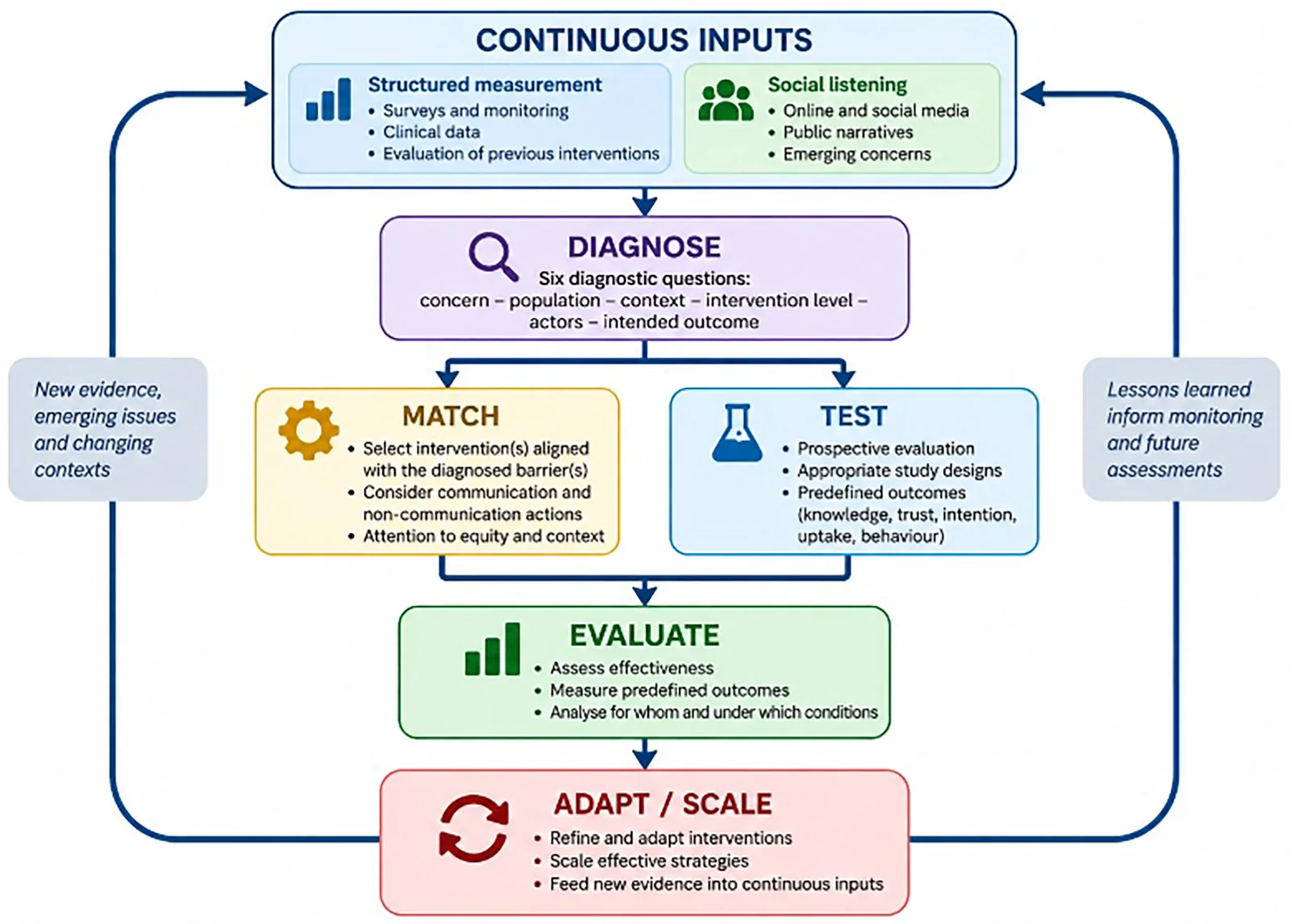
Diagnosis-driven framework for fluoride communication research and practice. Structured measurement and social listening provide continuous inputs to six diagnostic dimensions. Responses are then matched to the diagnosed barrier, prospectively tested, evaluated against predefined outcomes, and adapted or scaled, with findings feeding back into subsequent measurement and listening.

### 2.2 Refusal does not reveal the underlying mechanism

Observed refusal is an endpoint, not a diagnosis. The same behaviour may arise from different combinations of beliefs and circumstances, while similar concerns may produce different behaviours in different people. This distinction is important clinically and methodologically. Communication research should therefore separate at least three levels: the underlying concern or determinant; the expressed stance, such as hesitancy or opposition; and the behavioural outcome, such as acceptance, delay or refusal. Collapsing these levels into a single category weakens both explanatory research and intervention design.

Communication should therefore begin by identifying the barrier, the person or population experiencing it, the setting in which it is reinforced, and the outcome that an intervention is intended to change.

## 3 What vaccination research can and cannot teach oral health

### 3.1 Overlapping preventive behaviors

Vaccination research offers a valuable methodological precedent as it has progressed from describing hesitancy to measuring determinants, tailoring interventions and evaluating outcomes. Dentistry initially looked to this field for an empirical reason. In a study of 1,024 caregivers, immunisation refusal was associated with topical fluoride refusal, suggesting that preventive-care refusal behaviors can cluster within individuals or families (3). This finding justified asking whether the two domains might share attitudes and determinants.

Subsequent evidence made the picture more nuanced. Carpiano and Chi found that analogous attitudes toward childhood vaccines and topical fluoride could be correlated across domains, while their adjusted associations with actual refusal differed: some measures predicted vaccine refusal, whereas none of the analogous measures predicted topical fluoride refusal (4). A later study found that caregiver opposition to topical fluoride was associated with opposition to COVID-19 vaccination, showing that the behavioral association persisted in a different vaccination context (5).

### 3.2 Similar behavior does not imply identical determinants

The most important lesson is therefore not that fluoride hesitancy is a dental form of vaccine hesitancy. It is that similar preventive behaviors can have different patterns of determinants. Recent evidence illustrates this directly. Caregiver political affiliation was associated with childhood vaccine hesitancy, whereas it was not significantly associated with opposition to topical fluoride or community water fluoridation (6). The association of a given determinant with preventive attitudes or behaviors may therefore differ across prevention domains.

This distinction should shape the use of vaccination research in oral health. Vaccination research provides a methodological roadmap, not a causal template. Its principal value lies in methods - defining constructs, measuring drivers, segmenting populations, tailoring interventions and evaluating outcomes - rather than in assuming that specific determinants or messages can be transferred unchanged. Fluoride communication requires its own empirical diagnosis.

## 4 From description to measurement

### 4.1 Making topical fluoride hesitancy measurable

Progress requires constructs that can be measured reproducibly. The Fluoride Hesitancy Identification Tool (FHIT) was developed as a 20-item psychometrically evaluated measure of five domains of caregiver topical fluoride hesitancy: perceived lack of necessity, concern about chemicals entering the child's body, perceived harmfulness, uncertainty about topical fluoride science, and distrust or feeling pressured by dentists and their motives (7). The importance of the FHIT is not that it supplies a universal taxonomy, but that it converts an otherwise vague construct into dimensions that can be compared, tested and related to outcomes.

At the same time, existing evidence is concentrated in specific populations and settings. The FHIT should therefore be viewed as an important starting point for measurement rather than as a definitive or universally applicable representation of fluoride hesitancy. Cross-cultural validity cannot be assumed. Translation should involve more than language equivalence: the salience, meaning and structure of hesitancy domains may differ across countries, health systems, cultures and patterns of fluoride exposure. Measurement should therefore be treated as an empirical task rather than as a one-time instrument-selection decision.

### 4.2 Hesitancy and opposition are related but not identical

The first empirical application of the FHIT in a U.S. caregiver sample further clarifies the construct. Among 1,042 caregivers, 82.9% met the study definition of general hesitancy, while 39.1% expressed opposition to topical fluoride. Hesitancy was strongly associated with opposition, but the two constructs were not equivalent: 45.9% of hesitant caregivers expressed opposition, whereas 97.3% of those expressing opposition were hesitant (8). This asymmetry is conceptually important. Hesitancy can exist without opposition, and therefore should not be treated as a proxy for refusal or an “anti-fluoride” identity.

For communication practice, this creates an opportunity. Hesitancy may represent uncertainty, ambivalence or unresolved concerns rather than a fixed behavioural endpoint. For research, it means that studies should specify whether they are measuring beliefs, hesitancy, opposition, intention, uptake or refusal. These outcomes are related, but they are not interchangeable.

## 5 From measurement to diagnosis

### 5.1 Learning from vaccination behavioral science

Vaccination research has developed an increasingly structured progression from identifying behavioral and social drivers to tailoring and evaluating interventions. The WHO Behavioral and Social Drivers of Vaccination framework emphasizes systematic measurement and use of data to understand drivers and inform programme action (9). WHO and UNICEF's human-centered design approach explicitly links diagnosis with the design, implementation and evaluation of tailored immunization programmes (10). The ECDC social and behavioral science tools similarly emphasize identifying barriers and facilitators before designing context-specific strategies (11).

These frameworks should not be imported wholesale into fluoride communication. They nevertheless establish a methodological principle that is directly transferable: the intervention should follow diagnosis, not precede it. Fluoride research can adopt this progression while building domain-specific measures, evidence and implementation strategies. The six diagnostic dimensions proposed below were developed as a conceptual synthesis rather than adapted from a single existing model. They integrate fluoride-specific evidence on hesitancy and communication with methodological principles from the WHO Behavioural and Social Drivers framework, the WHO/UNICEF human-centred design approach and the ECDC social and behavioural science tools (9–11).

### 5.2 Structured measurement and social listening are complementary

Structured instruments are necessary because they provide standardized measures, comparable scores and the ability to monitor predefined constructs over time. Their strength is also their limitation: they measure what researchers already know to ask. A changing information environment can generate new narratives, vocabulary, concerns and information voids before these are represented in a questionnaire.

Social listening can complement structured measurement by analyzing questions, narratives, attitudes and uncertainties across multiple data sources, including health-system data, traditional media and user-generated digital and social-media content. WHO guidance describes social listening as a method for understanding conversations and concerns to inform proportionate and effective public-health responses (12). In fluoride communication, this function is particularly relevant because concerns may circulate across clinical encounters, peer networks, search engines, social platforms and, increasingly, AI-mediated interfaces. Potential risks of AI-generated health information may include amplification of inaccurate information, false certainty and opaque sourcing, while potential opportunities include improving access to understandable information, clarifying questions and directing users toward reliable sources. Because AI-mediated information environments are evolving rapidly, their role in shaping fluoride-related questions, source selection and representations of uncertainty should be treated as an empirical research question rather than assumed. The task is not to surveil disagreement, but to identify what people are asking, what remains unanswered and which narratives are gaining salience. Such approaches should be ethical, privacy-conscious and proportionate to the public-health purpose.

## 6 A diagnosis-driven framework for fluoride communication

The Authors propose a conceptual framework in which communication is treated as a researchable intervention rather than as an intuitive response. The framework is intended to organize diagnosis and intervention development; its utility, transferability and added value require prospective empirical evaluation. Two principles govern the model. First, structured measurement and social listening should operate continuously, because both the prevalence and the content of concerns may change. Second, a communication response should be selected only after six diagnostic dimensions have been specified.

### 6.1 Diagnostic core: six questions before choosing a response

The six diagnostic questions forming the core of the proposed framework are summarized in Table 1.

**Table 1:** Diagnostic core of the proposed framework.

Diagnostic question	Operational meaning
1. What is the concern?	Identify the dominant barrier or combination of barriers: perceived need or benefit, safety or dose, uncertainty, distrust or pressure, misinformation, or another context-specific concern. Example: dose.
2. Who is concerned?	Define the relevant population rather than assuming a generic public. Caregivers, patients, oral health professionals and communities may have different determinants and decision constraints. Example: caregivers of young children.
3. Where does the problem arise?	Locate the information and decision environment: chairside interaction, family and peers, community, search and social media, institutions, or AI-mediated interfaces. Example: a concern reinforced by social media before a dental visit.
4. What level of response is required?	Specify whether action is primarily individual, clinical, community-based, informational, organisational, public-health or policy-level. Example: clinical communication rather than population-wide messaging.
5. Who needs to be involved?	Identify credible messengers and necessary partners, including oral-health professionals, public-health authorities, behavioral scientists, communication specialists, professional organizations and communities. Example: the treating oral-health professional.
6. What outcome are the Authors trying to change?	Predefine the endpoint: knowledge, perceived benefit, risk perception, confidence, trust, intention, uptake, behavior or another measurable outcome. Example: change in risk perception rather than knowledge alone.

### 6.2 Action cycle: match, test, evaluate, adapt

Once the diagnostic core has been specified, the response enters an action cycle. Importantly, diagnosis may indicate that communication is not the appropriate intervention: some barriers may require changes in clinical practice, service organization, institutional transparency or policy rather than changes in messaging. When communication is indicated, the first step is Match: the strategy, messenger and setting should correspond to the diagnosed barrier. A perceived-benefit problem may require a different response from uncertainty; uncertainty requires a different response from distrust; and a specific misinformation narrative should not be treated as equivalent to general skepticism. The second step is Test: the selected response should be evaluated prospectively rather than assumed to work because it is scientifically accurate or intuitively persuasive.

The third step is Evaluate: success must be judged against the outcome defined before the intervention. Increased knowledge is not synonymous with increased trust; reduced risk perception is not synonymous with uptake; stated intention is not behavior. The fourth step is Adapt: ineffective components should be revised, effective components should be tested for transferability, and scaling decisions should consider context, feasibility and equity. For example, structured measurement or social listening might identify concern about fluoride harmfulness or dose among caregivers. Diagnosis would then specify the population, information setting and intended endpoint, such as risk perception or confidence. The matched response could use a trusted oral-health professional to explain dose and exposure, with the strategy prospectively compared with standard information. Evaluation would focus on the predefined endpoint rather than knowledge alone, and the response would be adapted or scaled only if effectiveness, context and equity support it.

### 6.3 Transparency, equity and participation are cross-cutting requirements

Two requirements apply across the entire cycle. The first is transparency. Communication should distinguish established evidence, legitimate uncertainty and unsupported claims, rather than implying certainty where uncertainty remains or treating every question as misinformation. The second is equity. A communication strategy that works in a well-resourced clinical setting may fail where access, language, digital connectivity, health literacy or continuity of care differ. Evaluating average effectiveness without examining distributional effects can therefore conceal widening inequalities.

These requirements also clarify the role of collaboration and participation. Researchers, oral health professionals, behavioral and social scientists, public-health authorities, communication specialists, patients and communities contribute different forms of evidence. Their involvement should not be viewed as a communication tactic added after the scientific work. It is part of the evidence infrastructure needed to define relevant questions, interpret findings, design feasible responses and ensure that research priorities reflect the populations and settings in which interventions will be implemented.

## 7 A research agenda for fluoride confidence

### 7.1 Transferability

Current measures and findings should be tested across countries, languages, cultures and fluoride-delivery settings. Cross-cultural validation should examine whether the same hesitancy domains retain their meaning and measurement properties, and whether additional context-specific domains emerge.

The framework should also be operationalized differently across fluoride-delivery contexts. Professionally applied topical fluoride involves an individual clinical decision, usually within a direct patient–professional or caregiver–professional interaction. Communication may therefore focus on perceived need, dose, safety, autonomy, trust in the clinician, and the immediate decision to accept or refuse treatment. Community water fluoridation operates at a population and policy level, where concerns may instead center on collective exposure, perceived control, institutional trust, equity, governance, and the legitimacy of public health decision-making. The same diagnostic framework can therefore be retained, but the relevant population, actors, setting, barriers, intervention level, and outcomes will differ according to the fluoride-delivery context.

### 7.2 Heterogeneity

Research should identify which concerns and determinants are shared across populations and which are context-specific or modifiable. This requires moving beyond prevalence estimates toward explanatory models that distinguish profiles of hesitancy and their relationships with opposition and behaviour.

### 7.3 Response

Communication and intervention strategies should be tested against specific diagnosed barriers. Comparative studies are needed to determine whether, for example, dose-and-exposure explanations, uncertainty communication, autonomy-supportive dialogue, prebunking or messenger changes work for different profiles rather than assuming a universally effective approach.

### 7.4 Outcomes

Studies should predefine the outcome they intend to change and avoid using knowledge as a default surrogate for trust, confidence, uptake or behavior. A mature evidence base will require outcome-specific effect estimates and clarity about whether short-term attitudinal change translates into sustained preventive behavior.

### 7.5 Methods and data

No single data source can capture the full phenomenon. Structured surveys, qualitative research, clinical interaction studies, social listening, longitudinal designs and intervention trials answer different questions. Their deliberate combination can connect individual-level determinants with changes in the broader information environment and provide a stronger basis for adaptive communication strategies.

Addressing these questions will require coordinated international research. Multicountry studies using harmonised core measures, while allowing for context-specific constructs, could distinguish determinants that are broadly shared from those that depend on culture, health systems, patterns of fluoride exposure and information environments. Such collaboration would also provide a stronger basis for testing whether effective responses are transferable across settings and can be scaled without widening inequalities. The proposed framework is conceptual and has not yet undergone prospective empirical validation. Its utility, transferability and measurement properties therefore require testing across populations, cultures, fluoride-delivery settings and health systems.

## 8 Conclusion

The well-established evidence base for fluoride prevention should be matched by greater methodological rigour in how fluoride confidence is studied and supported. Communication should not begin by selecting a message; it should begin by defining the problem. The proposed diagnosis-driven framework specifies the concern, population, information context, level of intervention, relevant actors and intended outcome before a response is chosen. Structured measurement and continuous listening inform this process, while responses are matched to diagnosed barriers, prospectively tested, evaluated against predefined endpoints and adapted or scaled according to effectiveness, context and equity.

The objective is not to persuade everyone to hold the same view about fluoride or to dismiss legitimate uncertainty as misinformation. Rather, it is to replace generic and reactive communication with transparent and evidence-based approaches. The next challenge is to determine what works, for whom, and under which conditions, and to make communication itself an object of scientific inquiry.

## References

[1] Chi DL Basson AA. Surveying dentists’ perceptions of caregiver refusal of topical fluoride. JDR Clin Trans Res. (2018) 3(3):314–20. 10.1177/2380084418761846 30938597

[2] Cruz S Ko A Chi DL. A qualitative study on dentists’ communication approaches in managing fluoride-hesitant caregivers. JDR Clin Trans Res. (2024) 9(3):212–20. 10.1177/23800844231203673 37908047 PMC11318380

[3] Chi DL. Caregivers who refuse preventive care for their children: the relationship between immunization and topical fluoride refusal. Am J Public Health. (2014) 104(7):1327–33. 10.2105/AJPH.2014.301927 24832428 PMC4056200

[4] Carpiano RM Chi DL. Parents’ attitudes towards topical fluoride and vaccines for children: are these distinct or overlapping phenomena? Prev Med Rep. (2018) 10:123–8. 10.1016/j.pmedr.2018.02.014 29755930 PMC5945909

[5] Saini SJ Carle AC Forsyth AR Chi DL. Association between caregiver opposition to topical fluoride and COVID-19 vaccines. Vaccine. (2023) 41(5):1035–41. 10.1016/j.vaccine.2022.12.018 36567141 PMC9747692

[6] Baldiotti ALP Chi DL. Caregiver political affiliation is associated with childhood vaccine hesitancy but not fluoride opposition. Vaccine. (2026) 89:128900. 10.1016/j.vaccine.2026.128900 42418961

[7] Carle AC Pallotto I Edwards TC Carpiano R Kerr DC Chi DL. Psychometric properties of the fluoride hesitancy identification tool (FHIT). PLoS One. (2024) 19(1):e0297188. 10.1371/journal.pone.0297188 38232098 PMC10793896

[8] Lim J Carle AC Carpiano RM Chi DL. Topical fluoride hesitancy and opposition are significantly and positively associated: a cross-sectional study. PLoS One. (2025) 20(4):e0322027. 10.1371/journal.pone.0322027 40305483 PMC12043125

[9] World Health Organization. Behavioural and Social Drivers of Vaccination: Tools and Practical Guidance for Achieving High Uptake. Geneva: World Health Organization (2022).

[10] World Health Organization, United Nations Children’s Fund. Human-centred Design for Tailoring Immunization Programmes. Geneva: World Health Organization and UNICEF (2022).

[11] European Centre for Disease Prevention and Control. Tools and Methods for Promoting Vaccination Acceptance and Uptake: A Social and Behavioural Science Approach. Stockholm: ECDC (2025).

[12] World Health Organization. Social Listening in Infodemic Management for Public Health Emergencies: Guidance on Ethical Considerations. Geneva: World Health Organization (2025).

